# *Herpes simplex* Virus Pneumonitis in an Acute/Subacute Paracoccidioidomycosis Patient With Malabsorption Syndrome. Case-Report and Literature Review

**DOI:** 10.3389/ffunb.2021.805502

**Published:** 2022-02-10

**Authors:** Ricardo S. Cavalcante, Bruno S. Souza, Iverson X. Duarte, Marcelo P. T. Moraes, Kunie I. R. Coelho, Beatriz L. Griva, Beatriz A. S. Pereira, Sueli A. Calvi, Marluci Betini, Rinaldo P. Mendes

**Affiliations:** ^1^Tropical Diseases Area, School of Medicine, São Paulo State University—UNESP, Botucatu, Brazil; ^2^Department of Pathology, School of Medicine, São Paulo State University—UNESP, Botucatu, Brazil; ^3^Nuclear Medicine Service, School of Medicine, São Paulo State University—UNESP, Botucatu, Brazil; ^4^University Library, São Paulo State University–UNESP, Botucatu, Brazil

**Keywords:** paracoccidioidomycosis, *Paracoccidioides brasiliensis*, paracoccidioidomycosis-herpetic infection comorbidity, *Herpes simplex* pneumonitis, herpetic infection and malnutrition, malnutrition

## Abstract

*Paracoccidioides* sp.—*Herpes simplex* virus (HSV) co-infection was not reported until now and malabsorption syndrome is a rare complication of the acute/subacute form (AF) of paracoccidioidomycosis (PCM), characterized by life-threatening abnormalities, such as fat and protein loss, lymphopenia, ascites, and intense immunosuppression. A 21-year-old woman presented the PCM AF with intense involvement of the abdominal and intestinal lymphoid organs, which leads to the malabsorption syndrome and severe immunosuppression. This patient developed a fatal-disseminated HSV infection associated with the paracoccidioidal disease. This case demonstrates that, in addition to the antigen-specific immunosuppression, some PCM patients can present a generalized cell-mediated immune depression and endogenous infection of latent microorganisms. On the best of our knowledge, this is the first report of an association between PCM and HSV infection.

## Introduction

Paracoccidioidomycosis (PCM) is a systemic disease caused by thermally dimorphic fungi of the *Paracoccidioides* genus that causes a wide range of clinical manifestations (Mendes et al., 2017) and an antigen-specific immunosuppression (Benard et al., 1996a).

Paracoccidioidomycosis acute/subacute form (AF) is a less prevalent (about 15%) clinical form, whose symptomatology is characterized by the involvement of the phagocytic-mononuclear system. It predominates in children, adolescents, and young adults, for which reason is also called “juvenile form” (Mendes et al., 2017). Only a few patients show the involvement of the abdominal lymphatic system and lymphoid organs, leading to malabsorption syndrome with protein loss, decreased peripheral blood lymphocyte count, chylous ascites, and humoral- and cell-mediated immune suppression (Bettarello et al., 1972; Andrade et al., 1976; Martinez et al., 1979a,b; Troncon et al., 1981). Such patients have shown opportunistic infections due to different infectious agents (Shikanai-Yasuda et al., 1992; Benard et al., 1996b). Because of the lack of information about this clinical picture, its diagnosis may delay, consequently, the treatment (Fernandes et al., 1988).

This case report presents a patient with the AF of PCM with abdominal lymph node enlargement and protein loss associated with a herpetic pneumonitis, a comorbidity not previously reported.

## Patients and Methods

### Case-Report

The case-report was written after analyzing the clinical files, laboratory data, image examinations, and autopsy findings of the patient.

This project was approved by the Research Ethics Committee of the São Paulo State University—UNESP Botucatu—School of Medicine, Brazil (protocol number 5,178,808).

### Literature Reviews – A. Systemic Mycoses and *Herpes simplex* Virus (HSV) Infection

The search was performed in BVS, LILACS, PubMed, Web of Science, COCHRANE, SCOPUS, and EMBASE until May 2019, using the strategy of the name of a systemic mycosis AND *Herpes simplex* infection (Table 1). (*B)* M*alnutrition and HSV infection*. The search was performed in EMBASE, CINHAL, COCHRANE, Web of Science, Scopus, and Lilacs until May 2020, using the strategy of malnutrition and similar names or terms AND *Herpes simplex* infection or similar (Table 1).

**Table 1:** Literature review search strategy list.

**Systemic mycosis × Herpes interactions**	**Malnutrition × Herpes interactions**
“Paracoccidioidomycosis” “Paracoccidioidal granuloma” “*Paracoccidioides brasiliensis* infection”	“Malnutrition” “Nutritional Deficiency” “Nutritional Deficiencies” “Undernutrition”
“Histoplasmosis” “*Histoplama* infection” “*Histoplasma capsulatum* infection”	“Malnourishment” “Malnourishments” “undernourishment”
“Blastomycosis” “*Blastomyces dermatitidis* infection” “Blastomycosis, South American” “Blastomycosis, North American” “Gilchrist Disease”	“*Herpes simplex* virus infection” “*Herpes smplex*”
“Coccidioidomycosis” “*Coccidioides immitis* infection” “Valley Fever” “San Joaquin Valley Fever”	
“Aspergillosis” “*Aspergillus* infection”	
“*Herpes simplex* virus” “*Herpes simplex”* “*Herpes labialis* virus” “*Herpes virus hominis”* “*Herpes-T* virus” “*Herpesvirus 1*, Saimiriine” “*Herpesvirus* 1 alpha, Saimirine” “*herpesvirus platyrhinae”* “*Herpesvirus* 16, Cercopithecine” “*Herpesvirus papio* 2” “Cercopithecine *Herpesvirus* 16” “Marmoset *Herpesvirus*” “Marmoset virus”	

## Results

The results are constituted by the case-report and the findings of the literature reviews.

### Case Report

A 21-year-old white housewife in previously good health was admitted on October 10, 2000 at the University Hospital—São Paulo State University—UNESP Botucatu—School of Medicine, Brazil with a 2-month history of progressive epigastric pain and a 7.0 kg weight loss. The patient also complained of cervical and inguinal lymph node enlargement without fistulization or pain for the past 20 days, as well as a temperature of 38–39°C, conjunctival and cutaneous jaundice, nausea, and vomiting with undigested food and biliary particles for the past 7 days. Her history revealed a 10-year-smoking habit-10 cigarettes/day, sporadic rural work, and a delivery in March 1998.

Physical examination revealed bad general condition, weight of 43.5 kg (usually 50.0 kg in woman), blood pressure of 100/60 mmHg, pulse rate of 102/min, respiratory rate of 31/min, axillary temperature of 37.6°C, conjunctival jaundice 3+/4+, lymph node enlargement in the cervical chains bilaterally and inguinal on the right side of tumoral type, and hepatosplenomegaly.

At admission, the patient showed anemia (8.8 g/dL hemoglobin and 27.1% hematocrit), normal platelets (298.10^3^/mm^3^), mild leukocytosis (12,500 cells/mm^3^), and normal lymphocyte count (2,000 cells/mm^3^). The results of the hepatic function tests, with normal values in parenthesis, were the following: ALT—alanine aminotransferase: 25 mIU/mL (21–75); AST—aspartate aminotransferase: 31 mIU/mL (17–59); alkaline phosphatase: 170 U/L (12–58); ⋎-GT—⋎-glutamyl transferase: 62 mIU/mL (15–73).

The product of fine-needle aspiration of a superficial lymph node demonstrated typical *Paracoccidioides* sp. yeast forms, confirming the PCM AF clinical picture. An abdominal CT scan performed on October 16, 2000, showed mild dilatation of intrahepatic biliary tract, enlarged lymph nodes—hepatic hilum, peripancreas, and retroperitoneum, diffusely increased pancreas with its heterogeneous head, heterogeneous hepatomegaly, homogeneous splenomegaly, and ascites. An endoscopic examination of the digestive tract on November 13, 2000, revealed hyperemic gastric mucous membrane, suggesting subepithelial hemorrhage, morbilliform exanthem in the gastric antrum and body. The histopathological examination of a biopsied tissue from gastric antrum and body was positive for *Helicobacter pylori*. The patient was treated with the association of omeprazole (40 mg orally every 12 h), amoxicillin (1.0 g orally every 12 h), and clarithromycin (500 mg orally every 12 h), during 7 days.

The serum concentration of each cytokine was determined in microplates sensitized with the specific monoclonal antibody, using a kit for ELISA (Genzyme Corp., Boston, MA, USA). The results indicated a Th-2 profile, with a 1,246 pg/mL TNF-α (normal 98 ± 25), 110 pg/mL interferon-γ (normal 214 ± 64), 20 pg/mL interleukin-2 (normal 103 ± 43), 31 pg/mL interleukin-4 (normal 10 ± 4), and 42 pg/mL interleukin-10 (normal 8 ± 6). The skin test with paracoccidioidin was negative on clinical and histopathological evaluations.

Although parenteral nutrition and erythropoietin were started on October 20, 2000 because of severe malnutrition and intestinal sub-occlusion, neither albumin and β-carotene serum levels nor hematologic parameters improved, and ascites increased.

Initial PCM treatment was carried out with intravenous amphotericin B every other day in increasing doses from 5.0 to 40.0 mg, reaching the total dose of 793 mg. The intestinal sub-occlusion responded to clinical measures.

### Course of the Disease

As the condition of the patient improved with amphotericin B, this patient was followed up as an outpatient with frequent readmissions for lack of compliance to antifungal treatment and nutritional support. A careful nutritional assessment was performed in every hospitalization. Diet was usually hypercaloric (1,800 kcal/day) and hyperproteic (1.4 g/kg body weight), with the administration of median-chain triglycerides. Her nutritional status always improved during hospitalizations, characterized by weight gain, but it worsened after every discharge.

At admission, this patient presented a weight of 43.5 kg, varying between 35.5 and 46.0 kg during follow-up.

Gallium and sulfur scans were carried out on January 18, 2001 revealing an inflammatory/infectious process in the cervical vertebra and hepatomegaly with indirect signs of hepatopathy.

**Figure 1:**
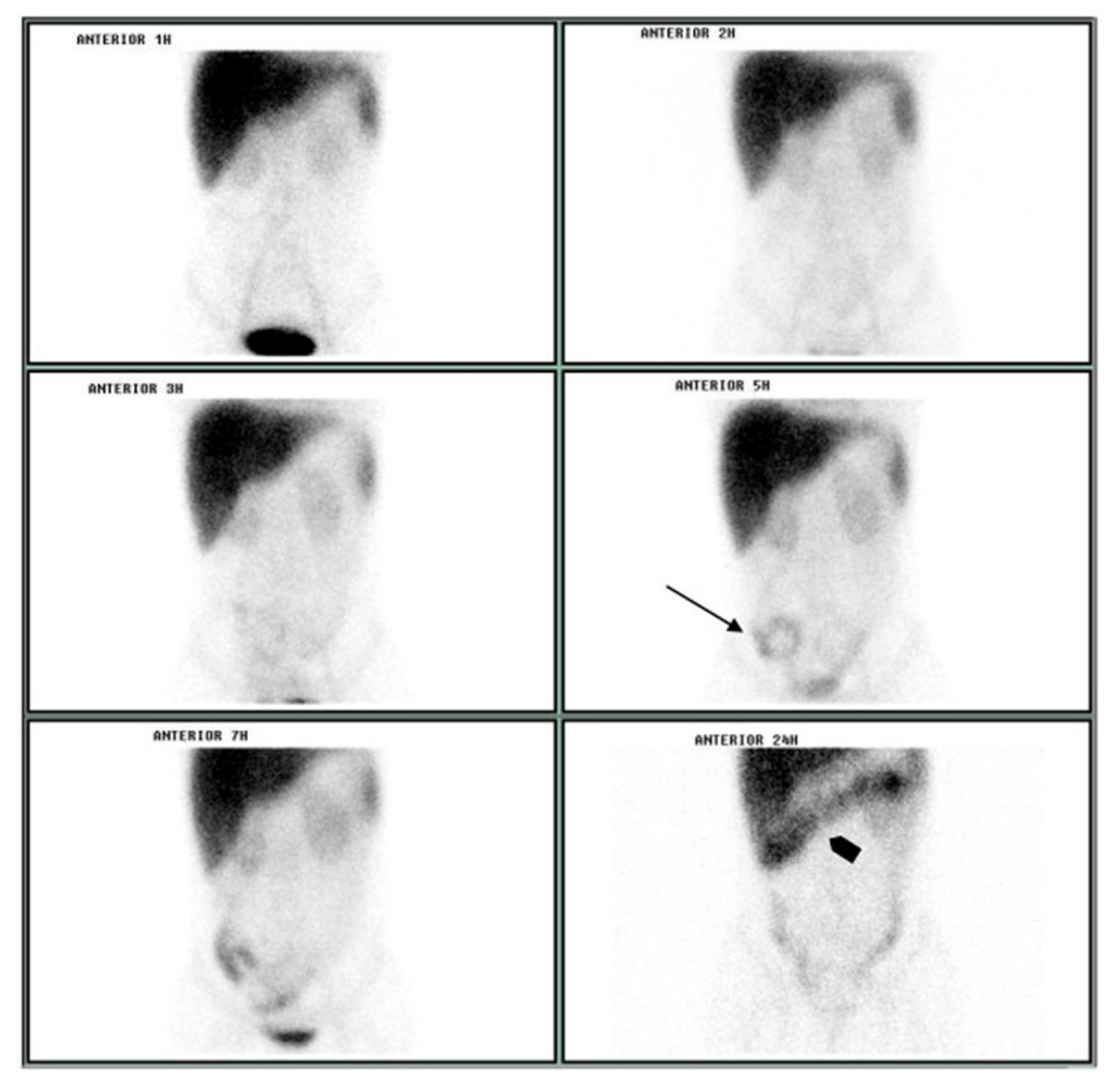
Sequential abdominal scintigraphy performed after the IV injection of ^99m^Tc-albumin: protein loss was unequivocally visualized on small bowel projection at 5 h image (arrow) with progression to colon at 24 h image (arrowhead).

Intestinal protein loss was confirmed on October 15, 2003 and the tests carried out on April 19, 2004 and on April 7, 2005. Urinary protein excretion was evaluated on October 15, 2003, showing 0.18 g/24 h (normal values between 0.04 and 0.23). Enteric protein loss was confirmed by sequential abdominal scintigraphy performed on October 15, 2003, after intravenously administered 555 MBq of ^99m^Tc-albumin by using a gamma-camera Orbiter (Siemens, Munich, Germany; Figure 1).

Two immunoenzymatic tests, ELISA bioMérieux^®^ and ELISA Abbott^®^ were carried out to investigate the HIV infection and resulted in non-reagent.

During the follow-up, hemoglobin and hematocrit increased, and platelets showed normal values until March 2006, when they decreased at her last hospitalization. Leukocyte count reduced after treatment, remaining in the lowest limit of normality. However, lymphocyte count (normal values 1,000–5,000/mm^3^) was in the lowest limit of normality for the first 3 years, this condition is called lymphopenia; it ranged between 2,352 cells.mm^−3^ during the first admission and 63 cells.mm^−3^ at the last hospitalization (average of 930 cells.mm^−3^ during the follow-up). Albumin serum levels (normal values 3.5–5.0 g/dL) were low at admission (2.3 g/dL) and decreased during almost all the follow-up, except for periods of readmissions and specific nutritional support, ranging from 2.7 to 0.9 g.dL^−1^ (average of 1.8 g/dL^−1^). The C-reactive protein showed increased values in almost all tests (73.3%), which were performed during the follow-up.

At her last admission, there was vomiting, pain in the right hypochondrium, bad general condition, weight of 36.6 kg, malnourishment, dehydration, blood pressure of 100/70 mmHg, pulse rate of 120/min, and respiratory rate of 28/min. Active PCM, intestinal occlusion, and pneumonia were diagnosed and treated. Nevertheless, this patient died on March 24, 2006, despite intravenous cotrimoxazole (trimethoprim-sulfamethoxazole combination, 240–1,200 mg 12/12 h), appropriate diet, antibacterial therapy (400 mg ciprofloxacin intravenously every 12 h and 500 mg metronidazole intravenously every 8 h), and general measures of support.

An evaluation of treatment compliance revealed a 76.5% attendance in 17 appointments to the PCM Outpatient Service, 40% attendance in 5 appointments in the Nutrition and Infection Outpatient Service, 36.4% antifungal compliance in 11 measures of serum sulfonamide levels, and a stable instead of descending serological curve, evaluated by the double-agar gel immunodiffusion test.

### Autopsy Findings

**Figure 2:**
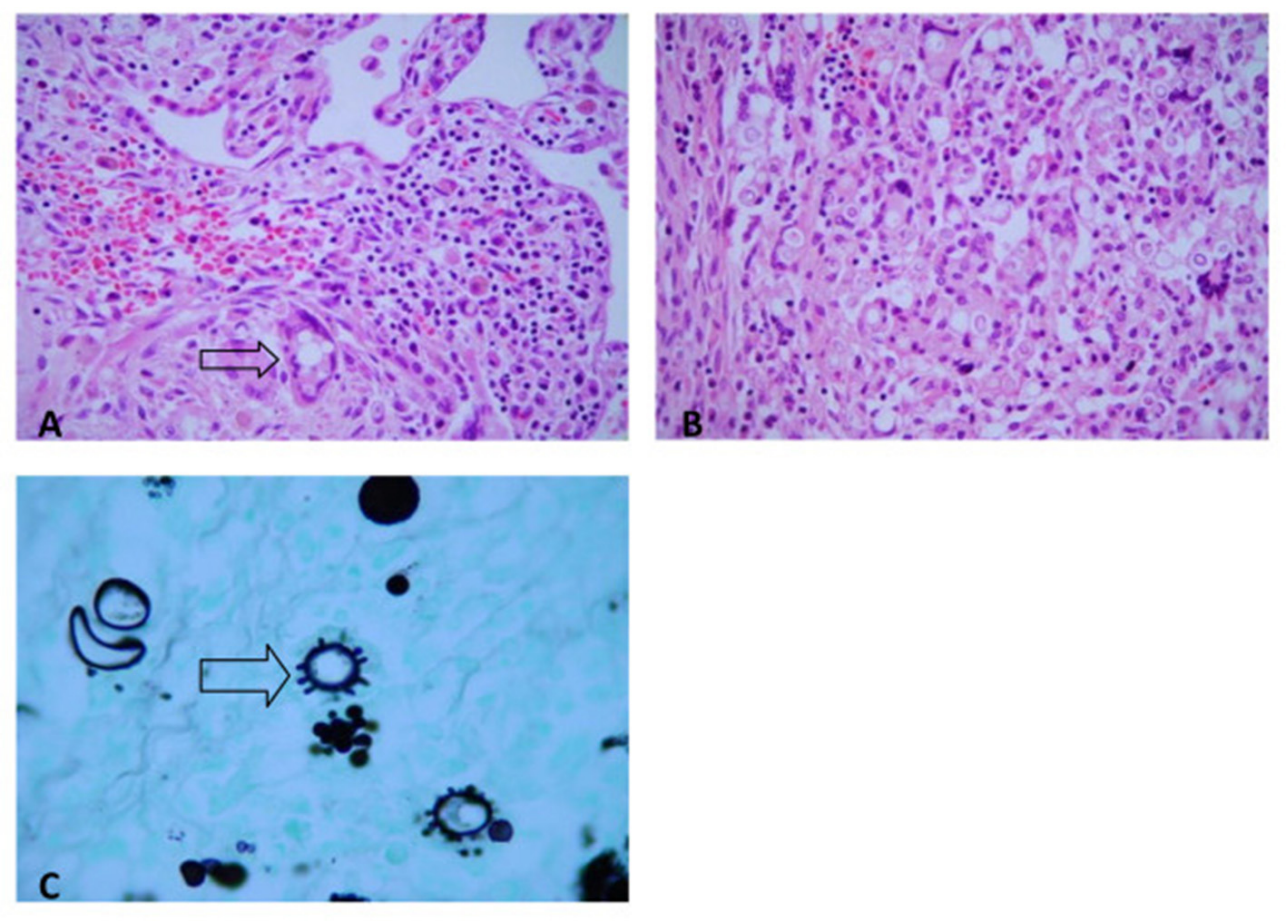
Paracoccidioidomycosis. **(A)** Lung. Epithelioid granuloma with multinucleated giant cells and yeast *P. brasiliensis* forms (H&E, ×200). **(B)** Colon. Epithelioid granuloma with multinucleated giant cells, and yeast *P. brasiliensis* forms (H&E, ×200). **(C)** Typical multiple-budding cell of *P. brasiliensis* seen in a GMS-stained (GMS, ×400).

At the autopsy, severe gross lesions in the spleen, terminal ileum, cecum, abdominal lymph nodes, pharynx, and larynx were observed. The spleen was enlarged (390 g) and presented numerous small whitish nodules which histologically corresponded to miliary granulomatous lesions of PCM. Terminal ileitis and typhlitis were represented by chronic ulcerative lesions with focal perforation and peritonitis; they were all active confluent PCM granulomatous lesions with central necrosis. Specific mycotic lesions were also severe in the pharynx and larynx; the lungs, liver, and bone marrow exhibited some discrete focal lesions (Figure 2).

**Figure 3:**
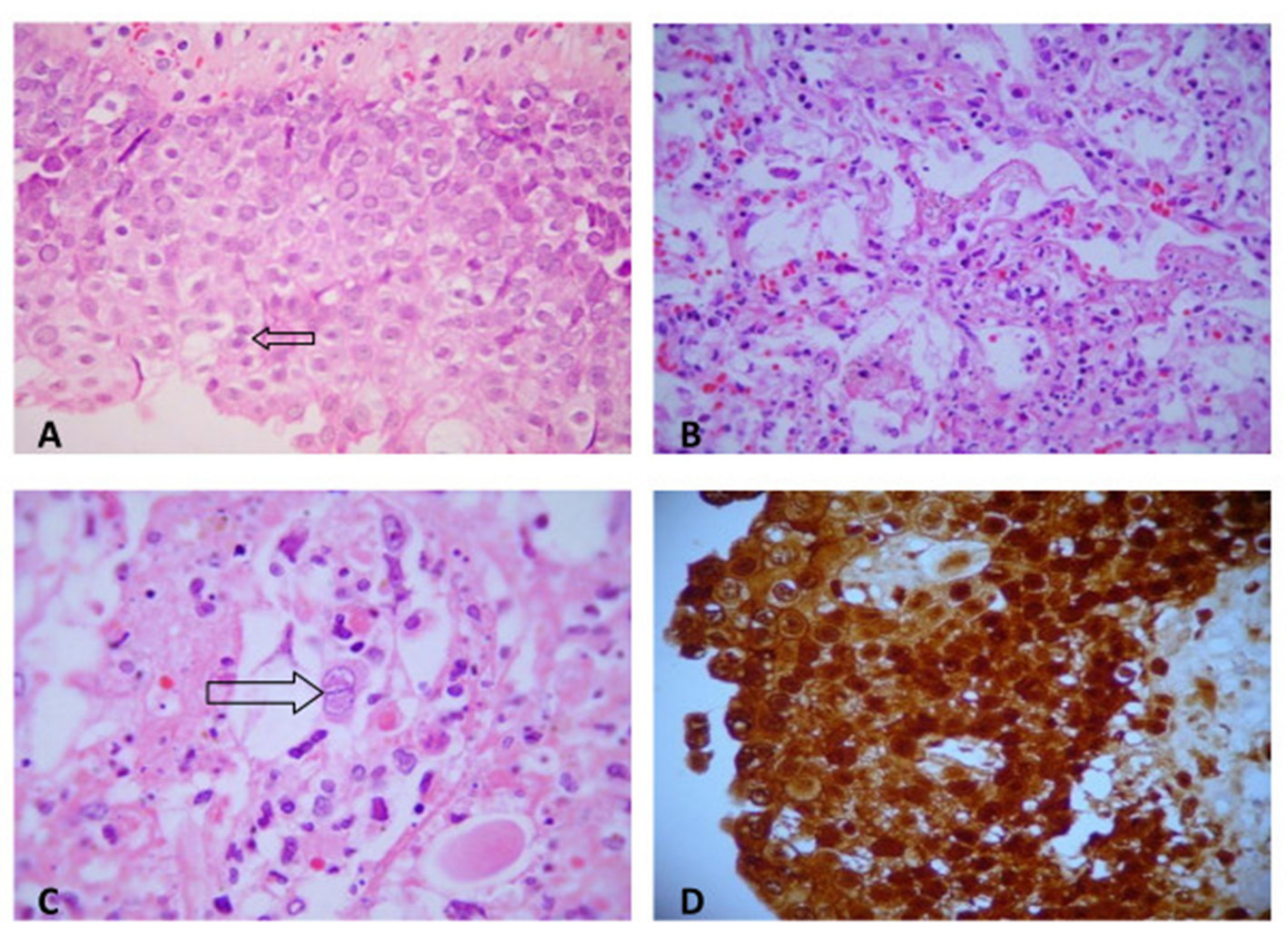
**(A)** Herpetic pharyngitis: numerous intranuclear viral inclusions in squamous epithelial cells (H&E, ×200). **(B)** Herpetic necrotizing pneumonitis (H&E, ×200). **(C)** Intranuclear herpetic inclusions in a pneumocyte (H&E, ×400). **(D)** *Herpes simplex* (immunohistochemistry). Numerous cells are strongly immunostained for herpes antigen within cytoplasma (peroxidase antiperoxidase stain, ×200).

Ulcerative pharyngitis and laryngitis besides extensive necrotizing pneumonitis were due to *Herpes simplex* infection. They presented characteristic intranuclear viral inclusions and were positive for *Herpes simplex* search by immunohistochemical staining (Figure 3).

### Literature Review—Systemic Mycosis—HSV Co-infection

Only two case reports were identified, involving AIDS as underlying disease and histoplasmosis as systemic mycosis.

First, a 51-year-old-patient of AIDS presented intense *Herpes simplex* oral infection associated with oral histoplasmosis and oral and systemic *Cytomegalovirus* (CMV) infection with fatal outcomes (Jones et al., 1992). At autopsy, CMV infection involved the oral mucous membrane, lungs, stomach, adrenal glands, and spleen, and was considered the primary cause of death, in addition to complications of AIDS. No evidence was found for the disseminated histoplasmosis or *Herpes simplex* infection was found.

Second, a 30-year-old–patient of AIDS with a confirmed cutaneous co-infection caused by HSV and *Histoplasma capsulatum* var. *capsulatum* (Méndez-Tovar et al., 2012). *H. capsulatum* was also identified in aspirate of bone marrow—confirmed diagnosis. Moreover, pulmonary interstitial infiltrates and increased serum levels of biochemical variables evaluating the liver were observed, constituting a presumptive diagnosis of organ involvement. As a whole, it is a case of cutaneous *Herpes simplex* infection and disseminated histoplasmosis. After the introduction of amphotericin B, followed by itraconazole and acyclovir, the patient improved. The follow-up, however, was lost because of immigration of the patient.

### Literature Review—Malnutrition—HSV Association

Only three articles were identified. An autopsy study evaluated 20 African patients with disseminated HSV infection, ranging in age from 2 to 48 months, 15 of them presented protein-energy malnutrition, 4 were post measles, and 1 showed no underlying disease (Raga et al., 1984). The second article reported six cases of generalized HSV infection associated with malnutrition, one of them presented also pulmonary tuberculosis (Templeton, 1970). The third article reported eight patients aged 9–16 months, seven of them with the signs of malnutrition, a finding emphasized by the authors (McKenzie et al., 1959).

## Discussion

The literature review showed only two cases of association between an agent of systemic mycosis and HSV infection (Jones et al., 1992; Méndez-Tovar et al., 2012). Both patients were HIV-infected, *H. capsulatum* was the fungus involved, and the herpetic infection was localized in one patient and disseminated in another. Thus, our case is, to the best of our knowledge, the first report of PCM associated with visceral active HSV infection, in an HIV-negative patient.

### *Herpes simplex* Virus Infection

Over 50 and 20% of the adult population show serological evidence of previous HSV types 1 and 2 infections, respectively (Koelle and Corey, 2003; Pollara et al., 2004). The host–HSV interaction varies from asymptomatic infection to severe diseases, such as herpetic encephalitis and fatal-dissemination, observed in immunosuppressed hosts (Fatahzadeh and Schwartz, 2007). Retrograde transport to the dorsal root ganglia (DRG) of the corresponding dermatome allows the virus to remain in a latent state for long periods. This latency depends on evasion strategies, such as restricted gene expression (Stevens, 1994), infection of tissues, and of cell types not readily accessible to the immune system (Barker and Billingham, 1977), suppression of MHC class I molecules for T-cell recognition, and interference with antigen presentation (Johnson and Hill, 1998). However, little is known about the mechanisms of reactivation or treatments that block this process (Halford et al., 1996).

Periodic reactivation at DRG can result from skin and/or mucosal lesions to wide disseminated disease, depending on the degree of viral replication, the number of DRG infected and reactivating, and the host immune response (Heng et al., 1994; Sasadeuz and Sacks, 1994; Langenberg et al., 1999).

*Herpes simplex* virus involvement of the adult lower respiratory tract (LRT) is rare and only few cases are part of a disseminated disease (Graham and Snell, 1983). The concomitant HSV involvement of the larynx, tongue, oropharynx, and esophagus supports Nash's hypothesis that LRT infection is a consequence of aspiration or contiguous spread (Nash and Foley, 1970; Nash, 1972).

Our patient showed concomitant ulcerative pharyngitis and laryngitis, and necrotizing pneumonitis, caused by HSV, suggesting that pneumonitis could be due to aspiration or contiguous spread, while the diffuse pulmonary involvement could have been caused by hematogenous spread (Ramsey et al., 1982), supported by previous findings of HSV viremia (Naraq et al., 1976) and the recovery of latent HSV from circulating lymphocytes (Nahmias and Roizman, 1973). However, the disseminated herpetic disease could show involvement of the brain or other visceral organs (Nahmias, 1970), which were not observed in our case. Our findings, taken together, make the decision about the migration route to the lung difficult.

As HSV classically infects squamous epithelium, it can be a possible explanation why a common pathogen is rarely described in the LRT (Graham and Snell, 1983). Among the factors that lead to squamous metaplasia is the smoking (Graham and Snell, 1983), a habit presented by our patients. The other possibilities are the pulmonary involvement by *Paracoccidioides* spp., observed in about 5% of the patients with the AF (Mendes, 1994) and/or the malnourishment favoring tissue invasion by HSV.

In patients with severe respiratory distress, HSV is the most frequently isolated pathogen from fiber-optic bronchoscopy specimens (Prellner et al., 1992), explaining the empiric treatment of bacterial pneumonia. HSV diagnosis can be demonstrated by cytological examination of appropriate respiratory samples since certain cytological findings are highly specific (Tuxen et al., 1982), by culture in human diploid fibroblasts or rabbit kidney cells—quick and more sensitive than the cytopathological examination (Nahmias and Norrild, 1979), by antigen detection in tissue by direct immunofluorescence staining and immunoperoxidase labeling—sensitive and specific methods (Taylor, 1978; Schmidt et al., 1980), although most of the patients cannot be submitted to a pulmonary biopsy. Serological methods are less invasive and easier to perform, but anti-HSV antibodies can be due to a previous infection (Xu et al., 2006); in addition, differently from primary infection, only 5% of the cases show a four-fold or higher increase of paired serum antibody titer at reactivation of latent foci.

### Acute/Subacute (Juvenile) Form and Intestinal Involvement in PCM

Our patient presented involvement of the intestines and abdominal lymphatic system as it has been observed in the AF of PCM. Although a malabsorption syndrome has rarely been demonstrated as a consequence of these lesions, it was present in our patient.

Intestinal lesions in PCM patients begin in the lymphoid follicles or agglomerations of lymphoid tissue of the submucosa, leading to follicular hypertrophy, which compresses the mucous membrane, protruding into the lumen and becoming macroscopically visible (Martinez et al., 1979a,b). Then, an expanding area of necrosis and lysis is observed and, after breaking the *muscularis mucosae*, it invades and destroys the mucosa, reaching the intestinal lumen and producing ulcerations. This process evolves with involvement of the mucous membrane and increased ulcerations, as it was observed in our case, sometimes with ileal perforation and granulomatous peritonitis.

Some patients show, adjacent to the granulomatous inflammatory reaction, a fibrotic neoformed tissue with vessels, fibroblasts, and sheaves of collagen fibers, which tend to become denser (Martinez et al., 1979a,b).

Spread of *Paracoccidioides* sp. yeast cells to the abdominal lymphoid system has been demonstrated by ultrasonography (Magalhães, 1980; Cerri et al., 1983), CT scan (Magalhães, 1980), and bipedal lymphography (Simão, 1975; Magalhães, 1980). Destruction of the lymph nodes, lymphatic hypertension, and obstruction of the lymphoid system in several levels was also observed (Simão, 1975). Such patients frequently reveal chylous ascites, due to the accumulation of lymph in the peritoneum, with high levels of protein and triglycerides, the latter being responsible for their opalescent aspect. However, carbohydrate malabsorption is rare.

Lymphatic stasis resulting from lymph node lesions causes chronic edema of the intestinal mucosa and leakage of lymph, rich in proteins and lymphocytes, to the digestive tract (Martinez et al., 1979a,b). Protein loss through mucosal ulcerations may be an associated mechanism (Martinez et al., 1979a,b).

Leakage of liquid to the extravascular territory leads to an imbalance of osmotic pressure and volume which, in turn, triggers renal mechanisms of water and sodium retention, thus maintaining ascites (Martinez et al., 1979b).

Immunoglobulin and lymphocyte loss frequently accompanies protein loss, such as albumin, lipoprotein, fibrinogen, transferrin, and ceruloplasmin, through the intestinal tract, in patients with malabsorption syndrome. Therefore, deficiency of humoral- and cell-mediated immunity is the usual outcome. Persistent low albumin serum levels substantiate the severity and irreversibility of these findings.

Our patient presented the AF (juvenile) of PCM, characterized by intense involvement of the phagocytic mononuclear system—lymph nodes, spleen, liver, and bone marrow. Bone lesions, usually asymptomatic, are frequently observed in these cases (Mendes, 1994). Gallium and sulfur scans showed a lesion in a cervical vertebra and hepatomegaly with indirect signs of hepatopathy, reinforcing its dissemination, and disease severity.

### Association Between Malnutrition and HSV Infection

The association between malnutrition and HSV infection was confirmed in neonates and children (McKenzie et al., 1959; Templeton, 1970; Raga et al., 1984). However, the PCM AF is also called the “juvenile form” (Mendes et al., 2017) because the clinical manifestations and the immunopathological profile are the same presented by children and adolescents. Thus, on the viewpoint of the immune response, our young adult patient answers like a child. In addition, some cases of infection with other microorganisms were reported in PCM-patients and attributed to the malnourishment due to malabsorption syndrome—*Cryptococcus* spp. (Shikanai-Yasuda et al., 1992; Benard et al., 1996b), bacterial, and mycobacterial agents (Shikanai-Yasuda et al., 1992).

### Pathogenesis

Our patient presented a past history of smoking habit, specific immunosuppression induced by the juvenile PCM form, malabsorption syndrome, disseminated PCM, and diffuse necrotizing pneumonitis caused by HSV. Taking these well-documented findings together, it is possible to suggest that the specific immunosuppression induced by fungi of the *Paracoccidioides* genus associated with the cell-mediated immunodepression caused by PCM-induced malnourishment led to the disseminated fungal disease and reactivation of latent HSV foci. The smoking habit might have favored the pulmonary lesions caused by HSV. It is not possible to deny an underlying immune defect that could predispose to both PCM and HSV infection, whose demonstration demands specialized approaches. The basic cause of this fatal outcome seems to be the malabsorption syndrome caused by the AF (juvenile form) of PCM, due to its severity, while the necrotizing pneumonitis by HSV seems to be a contributive factor. The autopsy findings support these conclusions.

### Patients Management

Reactivated HSV infection is rarely confirmed before death, mainly in the absence of labial or cutaneous lesions, as it occurred with our patient, whose diagnosis was neither suspected nor investigated.

As the course of disseminated herpetic infections is usually fulminant (Peters et al., 2000; Sevilla et al., 2004), some authors recommend its prevention with acyclovir (Peters et al., 2000; Dykewicz, 2001); this procedure was efficacious, although the mortality rate did not decrease, probably because of the underlying diseases (Tuxen et al., 1987).

In addition, serologic evaluation for the HSV infection should be performed in immunosuppressed patients, and acyclovir prophylaxis can be introduced in case of positivity, despite the poor prognosis (Peters et al., 2000; Dykewicz, 2001).

Our findings suggest the investigation of HSV infection in patients with respiratory distress syndrome caused by PCM, since most of these patients are cigarette smokers (Santos et al., 2003) and the prevalence of HSV-infection is high in the population (Xu et al., 2006). Depending on the results, acyclovir should be introduced.

## Data Availability Statement

The original contributions presented in the study are included in the article/supplementary material, further inquiries can be directed to the corresponding author/s.

## Ethics Statement

The studies involving human participants were reviewed and approved by Research Ethics Committee of the Faculdade de Medicina de Botucatu—São Paulo State University (Unesp). Written informed consent for participation was not required for this study in accordance with the national legislation and the institutional requirements.

## Author Contributions

RC and BS: medical assistance and data curation. ID, MM, and KC: investigation—histopathological findings. BG: investigation—nuclear medicine findings. BP and MB: investigation—systematic review. SC: investigation—immunological findings. RM: supervision, writing—review and editing, and medical assistance. All authors contributed to the article and approved the submitted version.

## Conflict of Interest

The authors declare that the research was conducted in the absence of any commercial or financial relationships that could be construed as a potential conflict of interest.

## Publisher's Note

All claims expressed in this article are solely those of the authors and do not necessarily represent those of their affiliated organizations, or those of the publisher, the editors and the reviewers. Any product that may be evaluated in this article, or claim that may be made by its manufacturer, is not guaranteed or endorsed by the publisher.
